# Thermal conductivity reduction of crystalline silicon by high-pressure torsion

**DOI:** 10.1186/1556-276X-9-326

**Published:** 2014-06-28

**Authors:** Sivasankaran Harish, Mitsuru Tabara, Yoshifumi Ikoma, Zenji Horita, Yasuyuki Takata, David G Cahill, Masamichi Kohno

**Affiliations:** 1 Department of Mechanical Engineering, Kyushu University, 744 Motooka, Fukuoka 819-0395, Japan; 2 Department of Materials Science and Engineering, Kyushu University, 744 Motooka, Fukuoka 819-0395, Japan; 3 International Institute of Carbon-Neutral Energy Research (WPI – I2CNER), Kyushu University, 744 Motooka, Fukuoka 819-0395, Japan; 4 Department of Materials Science and Engineering and Materials Research Laboratory, University of Illinois, Urbana, IL 61801, USA

**Keywords:** Silicon thermal conductivity, High-pressure torsion, Time domain thermoreflectance, Thermoelectrics

## Abstract

We report a dramatic and irreversible reduction in the lattice thermal conductivity of bulk crystalline silicon when subjected to intense plastic strain under a pressure of 24 GPa using high-pressure torsion (HPT). Thermal conductivity of the HPT-processed samples were measured using picosecond time domain thermoreflectance. Thermal conductivity measurements show that the HPT-processed samples have a lattice thermal conductivity reduction by a factor of approximately 20 (from intrinsic single crystalline value of 142 Wm^−1^ K^−1^ to approximately 7.6 Wm^−1^ K^−1^). Thermal conductivity reduction in HPT-processed silicon is attributed to the formation of nanograin boundaries and metastable Si-III/XII phases which act as phonon scattering sites, and because of a large density of lattice defects introduced by HPT processing. Annealing the samples at 873 K increases the thermal conductivity due to the reduction in the density of secondary phases and lattice defects.

## Background

Nanomaterials possess unique abilities to control thermal transport [1]. Engineering the thermal properties of nanostructured materials have a promising application in the field of thermoelectrics. The thermoelectric system performance is evaluated by the dimensionless figure of merit, ZT = *S*^*2*^*σT/k*, where *S* is the Seeback coefficient, *σ* is the electrical conductivity, *T* is the temperature, and *k* is the thermal conductivity [2]. To achieve higher ZT, lattice thermal conductivity of the thermoelectric material needs to be reduced without compromising the charge carrier mobility. Significant work has been done in recent years by using chemically distinct secondary phases either in the bulk form, or in the form of thin films, to reduce lattice thermal conductivity [3]. The introduction of nanostructured interfaces to scatter phonons efficiently and thereby reducing the thermal conductivity of the material has yielded high ZT in thermoelectric devices [4-6].

From an engineering perspective, practical thermoelectric device requires a significant volume of material. To realize this objective, nanostructuring using ball milling followed by hot pressing was shown to have a significant reduction in the thermal conductivity of thermoelectric materials especially silicon [7-10]. Similarly, mechanical deformation using high pressure was adopted to improve the Seeback coefficient of Bi_2_Te_3_ and PbTe [11,12].

Valiev et al. [13] demonstrated a novel technique using high-pressure torsion (HPT) to create a high density of lattice defects such as grain boundaries and dislocations on nanometer length scales [13]. Ikoma et al. [14,15], using HPT processing, reported detailed structural characterization of bulk crystalline silicon by X-ray diffraction spectroscopy, Raman spectroscopy, photoluminescence spectroscopy, and transmission electron microscopy and discussed the mechanism behind the nanograin formation in detail [14,15].

Intrinsic high thermal conductivity of single crystalline silicon limits its application in thermoelectric systems. In this work, we show that bulk single crystalline silicon, when subjected to intense plastic strain through HPT processing, shows a dramatic reduction in room temperature thermal conductivity from its intrinsic single crystal value of 142 W m^−1^ K^−1^ to a low thermal conductivity of approximately 7.6 W m^−1^ K^−1^. The experimental thermal conductivity results are comparable to nanostructured silicon prepared by ball milling and spark plasma sintering approach reported in the literature [7-10]. Considering the widely adopted method of ball milling followed by plasma sintering in thermoelectric literature to form bulk samples, the current approach could be a promising alternative for such applications.

## Methods

### Sample preparation

Single crystalline Si (100) wafers of size 5 × 5 mm^2^ and thickness 640 μm were subjected to HPT processing. Details of the HPT processing in Si was described elsewhere^14^. Briefly, the HPT facility comprises of upper and lower anvils made of tungsten carbide with flat bottomed spherical depressions to mount the test sample. During experiments, the test samples were placed in the lower anvil and the pressure was applied on the upper anvil. The HPT facility was operated at a pressure of 24 GPa (loading time approximately 7 s and unloading time approximately 2 s) and at room temperature. Torsional straining is achieved by rotating the lower anvil with respect to the upper anvil at a rotation speed of 1 rpm. HPT-processed samples with 0, 10, and 20 torsion cycles were prepared using this process. The samples were further annealed at 873 K for 2 h (0 and 10 torsion cycles) and 3 h (20 torsion cycles) in nitrogen atmosphere. We performed Raman and X-ray diffraction characterization independently and found that the experimental results were similar to previous literature results [14,15].

### Thermal conductivity measurements

**Figure 1:**
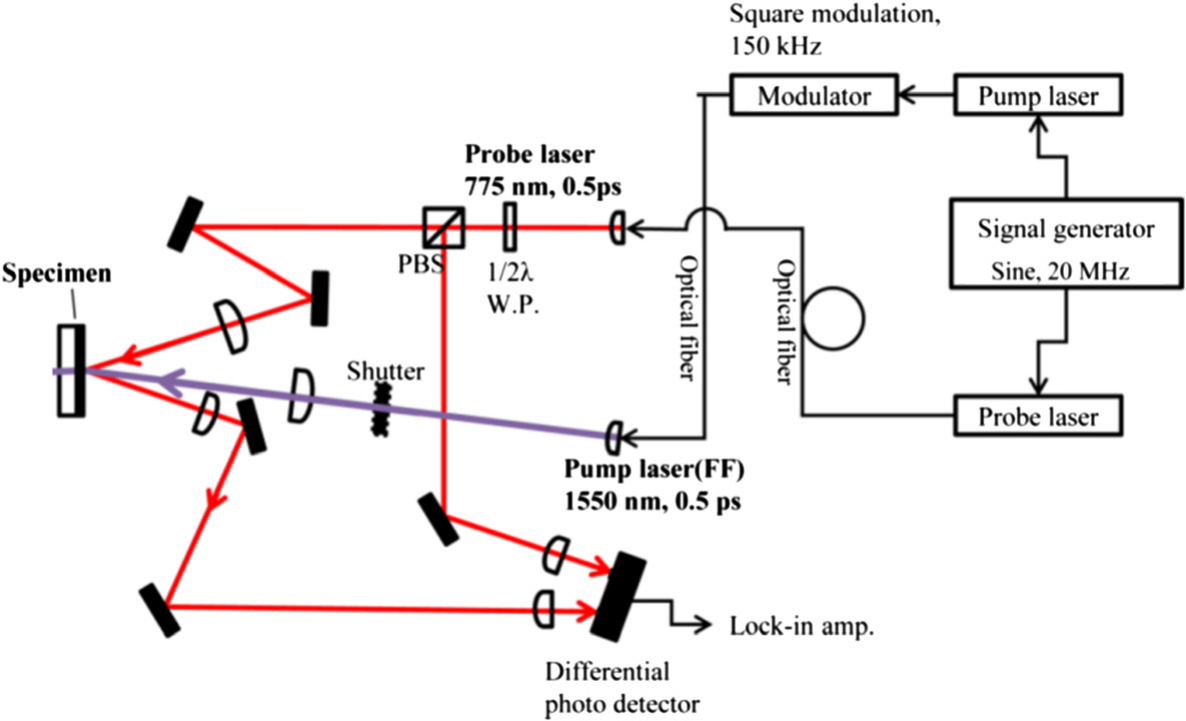
**Schematic of the picosecond time domain thermoreflectance setup.** The violet and red lines show the optical transport path of the pump beam and probe beam, respectively.

The thermal conductivity of HPT-processed samples were measured using picosecond time domain thermoreflectance (TDTR). Figure 1 shows the schematic of the TDTR experimental setup used in this study (Manufacturer - PicoTherm, Ibaraki, Japan). The output of the Er-doped fiber laser has a repetition frequency of 20 MHz. The pump beam of wavelength 1,550 nm heats the surface of a 135-nm-thick optothermal Al transducer film deposited on the sample by sputtering. The pump beam thermally excites the sample creating a temperature-dependent reflectivity change. The reflectivity change is separately monitored with a time-delayed probe laser of wavelength 775 nm. The in-phase component (*V*_in_) and the out-of-phase component (*V*_out_) of the probe signal variations were measured using a photodiode detector and audio frequency lock-in at 150 kHz.

The signals were analyzed assuming a unidirectional heat flow thermal model between the Al transducer film and the material [16]. In brief, the analysis model accounts for thermal transport in layered structures from time periodic power source with a Gaussian intensity distribution [17]. In our experiments, the modulation frequency of the pump beam is 150 kHz. The pump and probe beam spot sizes (1/e^2^ radius) are 37 μm and 14 μm, respectively. The Al transducer film thickness was measured as 135 nm using a profilometer.

## Results and discussion

**Figure 2:**
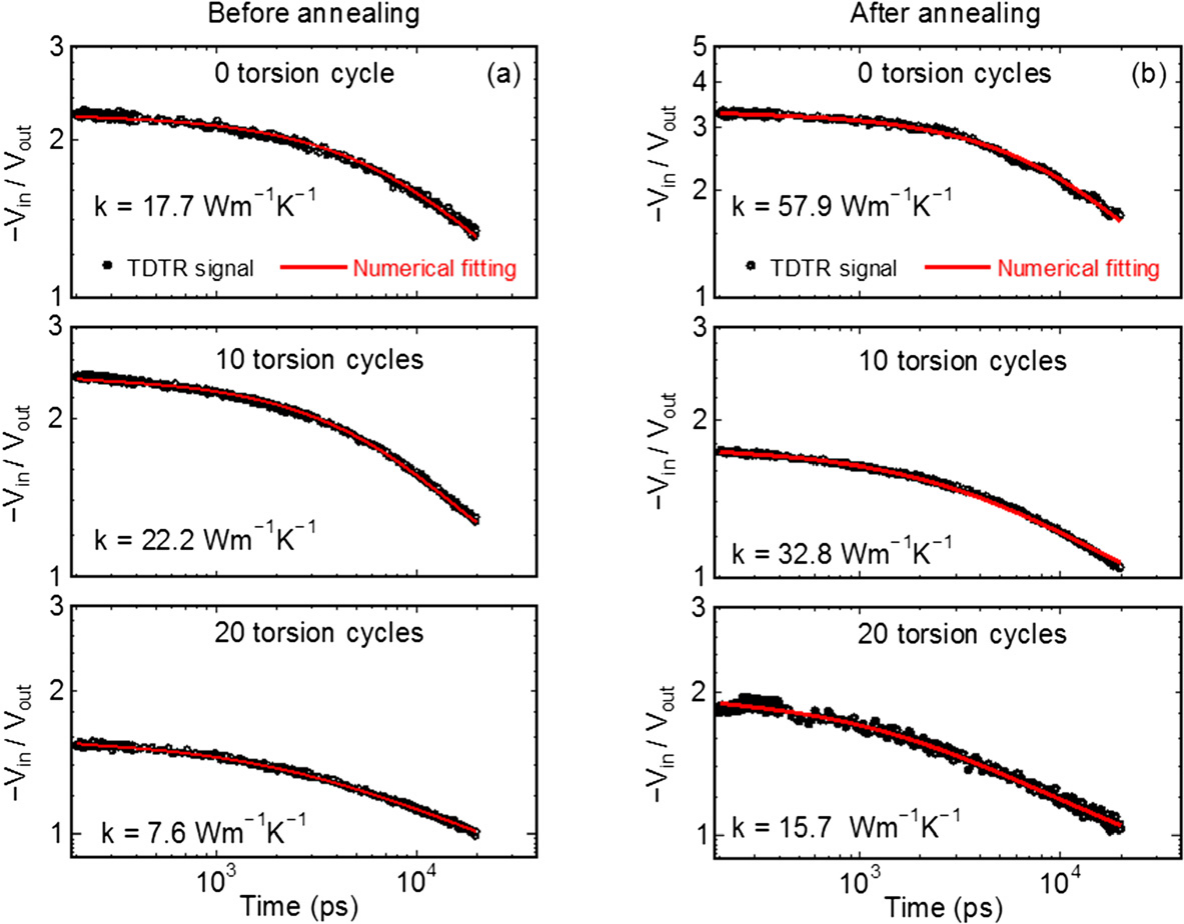
Example data set of HPT-processed sample and corresponding fitting of thermal model (a) before and (b) after annealing.

The thermal conductivity of single crystalline silicon with the Al transducer film was measured using TDTR and is found to be consistent with the literature value [18] within the experimental uncertainties of ±10%. The results of thermal conductivities of the HPT-processed samples measured using TDTR are shown in Figure 2. Figure 2a,b shows the example data sets and the corresponding numerical fitting to the thermal model. The free parameters used in the model, the thermal interface conductance of the Al/sample and thermal conductivity of the HPT sample are adjusted to fit the experimental data at different delay times.

**Figure 3:**
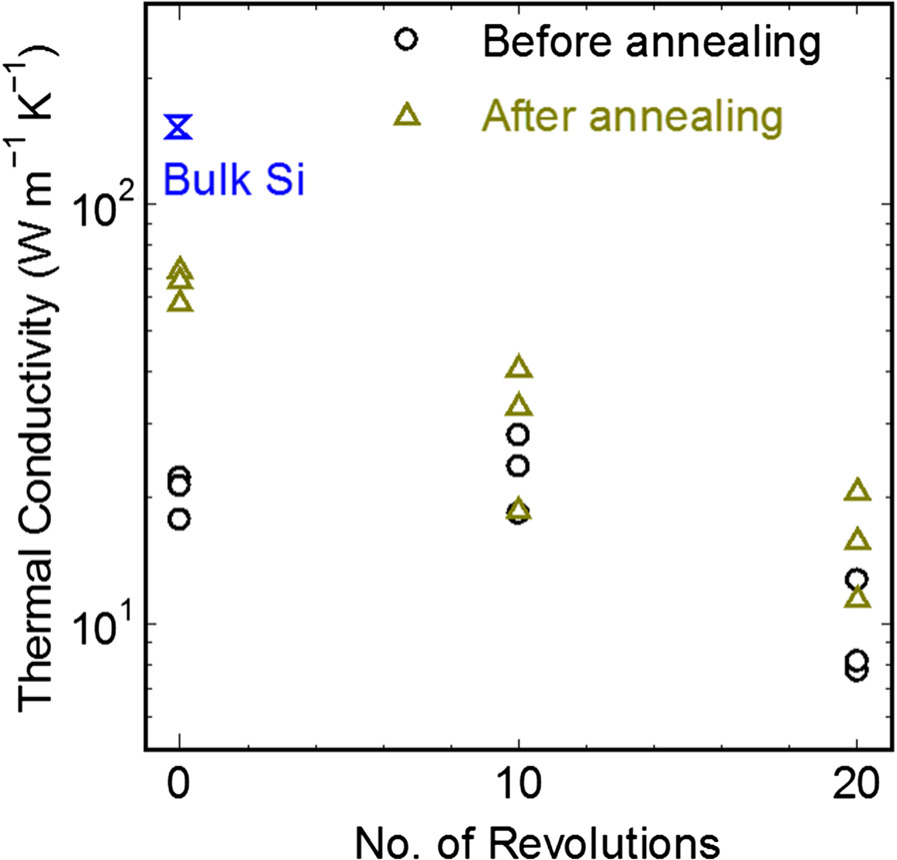
**Thermal conductivities of the HPT-processed before and after annealing.** An order of magnitude reduction in the thermal conductivity of Si upon HPT processing is observed. Annealing of the HPT samples show an increase in thermal conductivity due to the reverse transformation of metastable phases to Si-I cubic diamond phase.

Figure 3 shows the thermal conductivity results of the HPT-processed silicon before and after annealing. The thermal conductivity of the HPT-processed silicon at 24 GPa was approximately 18 Wm^−1^ K^−1^ which is an order of magnitude less than the intrinsic literature value of 142 Wm^−1^ K^−1^ for single crystalline silicon. The thermal conductivity of HPT-processed samples reduces to approximately 7.6 Wm^−1^ K^−1^ when further strained by HPT processing.

A possible reason for the dramatic reduction in lattice thermal conductivity is due to the decrease in grain size upon increasing plastic deformation. Our previous TEM investigations reported that the grain size of HPT samples reduces to as low as 10 nm during the HPT processing [14,15]. Hao et al*.*[19] theoretically calculated the thermal conductivity of nanograined silicon and showed that the thermal conductivity can be reduced to as low as 3 Wm^−1^ K^−1^ for a grain size of 10 nm which is comparable to the present experimental results. Phonon scattering at the nanograin boundaries increases as the grain size decreases which leads to the large reduction in the thermal conductivity. In addition, the presence of metastable Si-III/XII phases [14,15] creates lattice mismatch which further scatters the acoustic phonons. Based on the literature, it is anticipated that the thermal conductivity decreases with decreasing grain size. The present experimental results show that the mean thermal conductivity of 10 torsion cycle case (lower grain size) is marginally higher than the 0 torsion cycle case (higher grain size). The reason behind this deviation is still unclear. Nevertheless, the experimental results clearly show an order of magnitude reduction in thermal conductivity upon HPT processing.

Annealing of the HPT-processed samples results in an increase of thermal conductivity especially for the 0 torsion cycle case. The effect of annealing becomes less pronounced for the 10 torsion cycles (33 Wm^−1^ K^−1^ after annealing) and 20 torsion cycles sample (16 Wm^−1^ K^−1^ after annealing) resulting in a smaller increase in thermal conductivity. The increase in thermal conductivity is due to the reverse transformation of the metastable phases to Si-I diamond phase and also due to reduction in the density of lattice defects such as vacancies, dislocations, and grain boundaries. Since our previous study reveals that no appreciable grain coarsening occurs during the annealing process [14,15], the increase in thermal conductivity can be largely attributed to the reduction of the number of lattice defects; a contribution may also be present from the reverse transformation of metastable phases during annealing.

The present experimental results are comparable with the previous investigations in heavily doped p-type and n-type silicon. Existing literature results report a thermal conductivity reduction from approximately 100 W m^−1^ K^−1^ to 5 to 10 W m^−1^ K^−1^ at room temperature by varying the nature of alloy and the alloy concentration [7-10,20]. The alloy typically used is germanium and the samples are prepared by ball milling for several hours to achieve small nanograin structures followed by hot pressing at a temperature of 1,473 K to form a bulk sample [7-10]. The advantage of present technique over ball milling lies in the fact that bulk samples are directly prepared in a short duration by HPT processing to obtain a dramatic reduction in thermal conductivity by a factor of approximately 20. Although, the present thermal conductivity of approximately 7.6 Wm^−1^ K^−1^ is still high for thermoelectric application, we anticipate that by using HPT processing combined with appropriate doping will result in further reduction of thermal conductivity of silicon and possibly other thermoelectric materials such as SiGe, Bi_2_Te_3_, and PbTe.

## Conclusions

In summary, we demonstrated a novel way to reduce the lattice thermal conductivity of crystalline silicon by intense plastic strain through high-pressure torsion (HPT) at a pressure of 24 GPa. The grain boundary size decreases to nanoscale levels upon increasing the strain by HPT processing. The thermal conductivity of the HPT samples decreases to as low as approximately 7.6 Wm^−1^ K^−1^ due to the increase in phonon scattering at the nanograin boundaries. The present results introduce an efficient and irreversible way to make nanograin boundaries and provide a potential tool for the fabrication of thermoelectric materials with improved performance.

## Competing interests

The authors declare that they have no competing interests.

## Authors' contributions

SH and MT together performed the thermal conductivity measurements and drafted the manuscript. YI and ZH prepared the silicon samples for thermal measurements. DGC supervised the data analysis and interpretation of the results. YT and MK conceived the idea and supervised the project. All authors read and approved the final manuscript.
